# Sex Differences in Fatty Acid Metabolism and Blood Pressure Response to Dietary Salt in Humans

**DOI:** 10.3390/cardiogenetics13010005

**Published:** 2023-03-03

**Authors:** Jeanne A. Ishimwe, Jane F. Ferguson, Annet Kirabo

**Affiliations:** 1 Division of Clinical Pharmacology, Department of Medicine, Vanderbilt University, Nashville, TN 37235, USA; 2 Division of Cardiovascular Medicine, Department of Medicine, Vanderbilt University, Nashville, TN 37235, USA; 3 Vanderbilt Translational and Clinical Cardiovascular Research Center, Nashville, TN 37235, USA; 4 Medical Center, Department of Molecular Physiology and Biophysics, Vanderbilt University, Nashville, TN 37235, USA

**Keywords:** salt sensitivity, hypertension, sex differences, high salt, metabolomics, fatty acids, inflammation

## Abstract

Salt sensitivity is a trait in which high dietary sodium (Na^+^) intake causes an increase in blood pressure (BP). We previously demonstrated that in the gut, elevated dietary Na^+^ causes dysbiosis. The mechanistic interplay between excess dietary Na^+^-induced alteration in the gut microbiome and sex differences is less understood. The goal of this study was to identify novel metabolites in sex differences and blood pressure in response to a high dietary Na^+^ intake. We performed stool and plasma metabolomics analysis and measured the BP of human volunteers with salt intake above or below the American Heart Association recommendations. We also performed RNA sequencing on human monocytes treated with high salt in vitro. The relationship between BP and dietary Na^+^ intake was different in women and men. Network analysis revealed that fatty acids as top subnetworks differentially changed with salt intake. We found that women with high dietary Na^+^ intake have high levels of arachidonic acid related metabolism, suggesting a role in sex differences of the blood pressure response to Na^+^. The exposure of monocytes to high salt in vitro upregulates the transcription of fatty acid receptors and arachidonic acid-related genes. These findings provide potentially novel insights into metabolic changes underlying gut dysbiosis and inflammation in salt sensitivity of BP.

## 1. Introduction

The 2022 update on heart disease and stroke statistics by the American Heart Association reports that 47% of the US adult population have hypertension [1]. Excess dietary salt is a major risk factor for hypertension and cardiovascular disease [2]. The American Heart Association recommends 2300 milligrams (mg) of Na^+^ intake daily, but less than 10% of the US population follows this recommendation. Salt-sensitivity of blood pressure (SSBP), defined as a 10% increase in blood pressure in response to sodium (Na^+^) load, affects 50% of the hypertensive and 25% of the normotensive population. The detrimental role of high salt is further supported by strong evidence that a reduction in dietary Na^+^ lowers blood pressure and reduces annual new cases of coronary heart disease and stroke in the US by 20% [3]. The lack of compliance with dietary salt guidelines is particularly alarming because high salt intake increases the risk of mortality in salt-sensitive individuals, even independent of hypertension. Although hypertension is more prevalent in males, its development across the lifespan is steeper in females [4]. Population studies show that women are more salt-sensitive than men regardless of age [5]. The mechanism by which women are more salt sensitive is not fully understood.

The kidneys play an important role in blood pressure regulation through the modulation of Na^+^ reabsorption mediated by transporters including the epithelial Na^+^ channel. Emerging evidence indicates extrarenal mechanisms in the pathogenesis of SSBP and associated sex differences in Na^+^ regulation mediated by the skin, skeletal muscle, and gut [6,7]. We previously demonstrated that, in the gut, elevated dietary Na^+^ alters the microbiome, prompting an increase in Firmicutes phylum and genus *Prevotella* bacteria [8]. The mechanistic interplay between excess dietary Na^+^-induced alteration in the gut microbiome, inflammation, and sex differences is less understood.

Chronic low-grade inflammation involving both the innate and adaptive immune systems plays a key role in the pathophysiology of hypertension and salt sensitivity. The inflammation in hypertension and other cardiovascular disorders does not typically present in some of the classical definitions of physical characteristics, including heat, pain, redness, and swelling. Instead, their activation mediates physiological changes in the function of organs. Immune cells, including dendritic cells and T cells, can infiltrate tissues and promote the development of hypertension by releasing cytokines that cause vascular dysfunction and tissue damage. This process can be initiated and enhanced by hypertensive stimuli including Na^+^, suggesting a direct link between excess Na^+^, inflammation, and salt sensitivity of blood pressure [9,10]. Adoptive transfer of splenic immune cells causes blood pressure changes, cementing that immune cells play a critical role in hypertension [11,12]. Na^+^ accumulates in tissues and promotes the induction of proinflammatory mechanisms including the T_H_17 pathway, the formation of immunogenic isolevuglandins, and inflammasome activation [11–14]. The activation of these pathways leads to the production of proinflammatory cytokines, including interleukin-1β and interleukin 6, which promote renal and vascular dysfunction and end-organ damage, resulting in hypertension [15]. Microbes in the gut produce inflammatory metabolites such as short-chain fatty acids and bile acids, further potentiating inflammation [16]. Thus, inflammation may be a mechanistic link between gut dysbiosis, gut-associated metabolites, and salt sensitivity of blood pressure. More studies are needed to elucidate the metabolomic dysregulation associated with high salt, salt sensitivity, and sex differences. We hypothesized that high dietary salt-induced dysbiosis is associated with distinct metabolomic changes in men and women. The goal of this study was to identify novel metabolic players in sex differences and blood pressure in response to a high dietary Na^+^ intake.

## 2. Materials and Methods

Human study population. We previously recruited 135 healthy volunteers as part of the ABO Glycoproteomics in Platelets and Endothelial Cells (ABO) study [8,17]. Participants were 44 nonpregnant/nonlactating women and 31 men. We excluded subjects who had a history of organ transplantation, use of tobacco, and prescription medication, with the exception of oral contraceptives. Blood pressure was measured and recorded using a non-invasive cuff and automated sphygmomanometer in individuals who were seated. Stool and plasma samples were collected to study the metabolome in a subset of 75 from the initial 135 cohort of volunteers.

Human dietary sodium estimation. Study participants were asked to pause consumption of over-the-counter medications, supplements, and vitamins for 2 weeks leading to sample collection. They completed a 3-day food record, which was analyzed using Food Processor 8.1 (ESHA Research) to estimate dietary Na^+^ consumption as previously described [8]. Based on the recommendation by the American Heart Association, participants were classified as normal (<2.3 g Na^+^/day) and high salt (≥2.3 g Na^+^/day) based on their estimated daily Na^+^ intake.

Metabolomics profiling. Metabolomics profiles of plasma and fecal samples were conducted at Metabolon (Metabolon Inc., Morrisville, NC, USA; global metabolomics platform). Fecal samples were processed as previously described [18]. Samples were prepared using the automated MicroLab STAR^®^ system from Hamilton Company. Several recovery standards were added before the first step in the extraction process for QC purposes. To remove protein, dissociate small molecules bound to protein or trapped in the precipitated protein matrix, and recover chemically diverse metabolites, proteins were precipitated with methanol under vigorous shaking for 2 min (Glen Mills GenoGrinder 2000) followed by centrifugation. The resulting extract was divided into five fractions: two for analysis by two separate reverse phases (RP)/UPLC-MS/MS methods with positive ion mode electrospray ionization (ESI), one for analysis by RP/UPLC-MS/MS with negative ion mode ESI, one for analysis by HILIC/UPLC-MS/MS with negative ion mode ESI, and one sample was reserved for backup. Samples were placed briefly on a TurboVap^®^ (Zymark) to remove the organic solvent. The sample extracts were stored overnight under nitrogen before preparation for analysis.

Metabolomic Analysis. Global metabolomics analysis was conducted using the web server MetaboAnalyst 5.0 [19,20]. Pathway correlation networks and discriminant analyses were conducted on plasma and stool samples. The novel Microbiome SmartPanel (Metabolon Inc., Morrisville, NC, USA; Microbiome SmartPanel platform) was used to investigate gut microbiome-related biochemical sex differences.

RNA Sequencing (RNAseq) Analysis. RNAseq was performed as previously described [7,11]. Briefly, we used Ficoll-gradient to isolate peripheral blood mononuclear cells (PBMCs) from heparinized blood collected from 11 study participants. Demographic information of the participants has been previously reported [7]. We isolated monocytes by magnetic labeling and negative selection using the Miltenyi monocyte isolation kit (Miltenyi Biotec, Cat# 130–091-151), according to the manufacturer’s protocol using LS columns. We cultured the monocytes in 12-well plates at 1 × 10^6^/mL density in either normal salt RPMI media (150 mM) or high salt RPMI media (190 mM) for 72 h. RPMI media 1640 (Gibco) was supplemented with 10% FBS, 1% pen/strep, 1% HEPES, and 2-Mercaptoethanol (0.05 mM). Total RNA was isolated using the RNEasy Midi Kit (Qiagen, Valencia, CA, USA) following the manufacturer’s protocol. Targeted analysis of polyadenylated transcripts was performed using the Illumina Tru-seq RNA sample prep kit and paired-end sequencing was performed on the Illumina HiSeq2500. Using the R package, the FASTQ data files from the paired-end sequencing analysis were aligned with TopHat 2 for each sample against the human GRCh38 reference genome assembly. Quality control for the RNAseq data occurred during the following stages: (1) RNA quality; (2) raw read data (FASTQ); (3) alignment; (4) gene expression. Quality control for the raw data and alignment were performed using QC3, and the MultiRankSeq method was used for expression analysis. Raw data false discovery rate (FDR < 0.05) was used to correct for multiple hypothesis testing.

Statistics. Statistical analyses were performed by GraphPad prism 9. Continuous data are expressed as mean ± SD. Relationships between continuous variables were assessed by Pearson correlation. Student’s *t* test was used to compare two groups. Means were considered significantly different if *p* < 0.05.

## 3. Results

### 3.1. Blood Pressure and Dietary Na^+^ Intake in Men and Women

**Figure 1.**
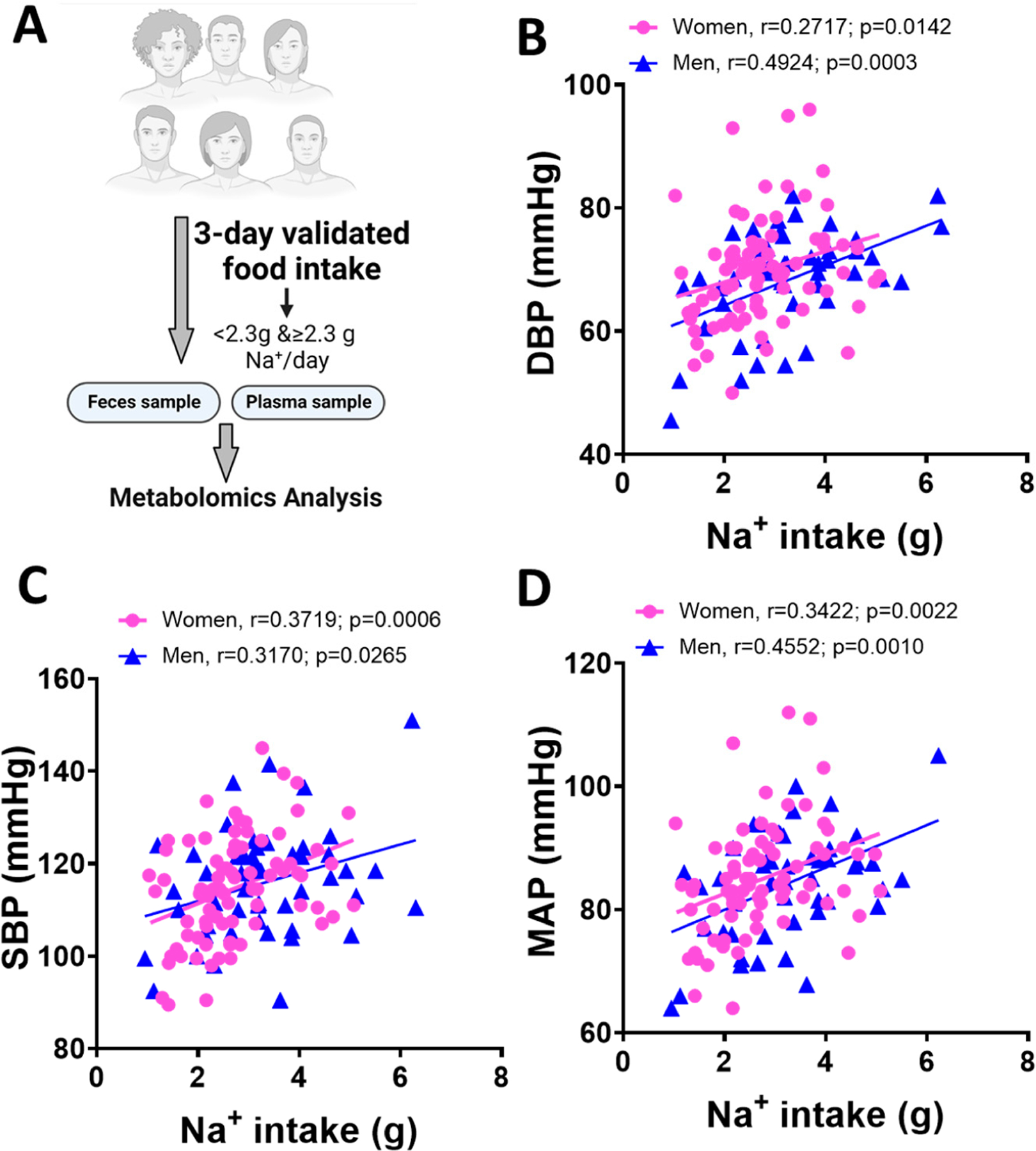
Relationship between a high-salt diet, sex differences and blood pressure. (**A**) Study design: self-reported short-term dietary Na^+^ intake was estimated from 3-day food records before the study visit. Metabolomic analysis was performed on plasma and fecal samples. (**B**) Diastolic pressure relationship with dietary Na^+^ intake. (**C**) Systolic pressure relationship with dietary Na^+^ intake. (**D**) Mean arterial pressure relationship with dietary Na^+^ intake in men (*n* = 31) and women (*n* = 44). Blue symbols represent men. Pink symbols represent women.

We assessed the relationship between the reported salt intake and blood pressure in men and women. Subjects were categorized into men and women based on their responses to the survey. We studied a cohort of 75 healthy volunteers who completed validated three-day food intake records (Figure 1A). The general characteristics of subjects in this study evaluating metabolomic changes are shown in Table 1. Men were older than women. They also had higher body mass index and larger waist circumference. As expected, men had significantly higher diastolic, systolic, and mean arterial pressure than women. We conducted Pearson’s correlation analysis to examine blood pressure to Na^+^ intake stratified by sex. This analysis was conducted on all 135 healthy volunteers in the ABO study whose demographic information has been previously reported [8] to increase power. We found a significant positive correlation between diastolic (Figure 1B, r = 0.2714 for women and r = 0.4924 for men), systolic (Figure 1C, r = 0.3719 for women and r = 0.3170 for men) and mean arterial pressure (Figure 1D, r = 0.3422 for women and r = 0.4552 for men) in both sexes.

### 3.2. Metabolomics Analysis Results

**Figure 2.**
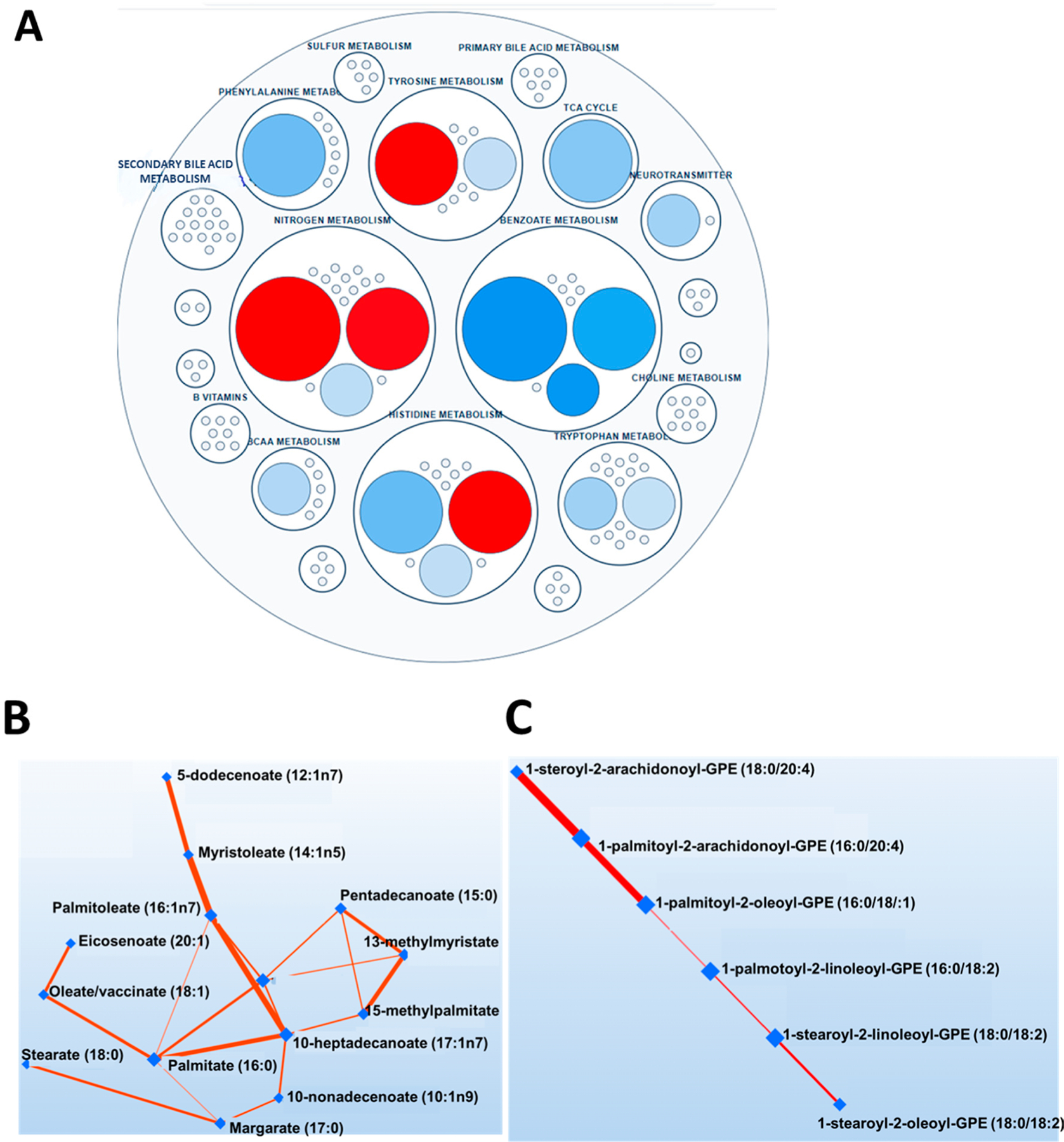
Top metabolic pathways in sex differences and a high-salt diet. (**A**) SmartPanel analysis shows significantly different pathways between men and women. (**B**,**C**) Pathway network analysis from a global metabolomic analysis identifying fatty acids as a top significantly changed pathway on a high Na^+^ diet. Men (*n* = 31) and women (*n* = 44).

Our global metabolomics analysis detected 801 metabolites in the plasma. To assess sex differences in global metabolites, we conducted a SmartPanel analysis and found 17 significantly different metabolites. Amounts of 1-methyhistamine, tyramine, cadaverine and N-acetyl-cadaverine were higher in men. On the other hand, hippurate, 3-hydroxyhippurate, benzoate, imidazole lactate, 3-hydroxyphenylacetate, tricarballylate, kynurenate, N-acetylaspartate, 2,3-dihyroxyisovalerate, glutamine, histidine, tyrosine and quinolinate were lower in men compared to women (Figure 2A). A Debiased Sparse Partial Correlation network analysis revealed fatty acids as top subnetworks differentially changed between normal and high salt groups (Figure 2B,C). Following our findings, that the metabolism of the fatty acids was the top pathway associated with high salt intake, we focused our subsequent analysis here.

**Figure 3.**
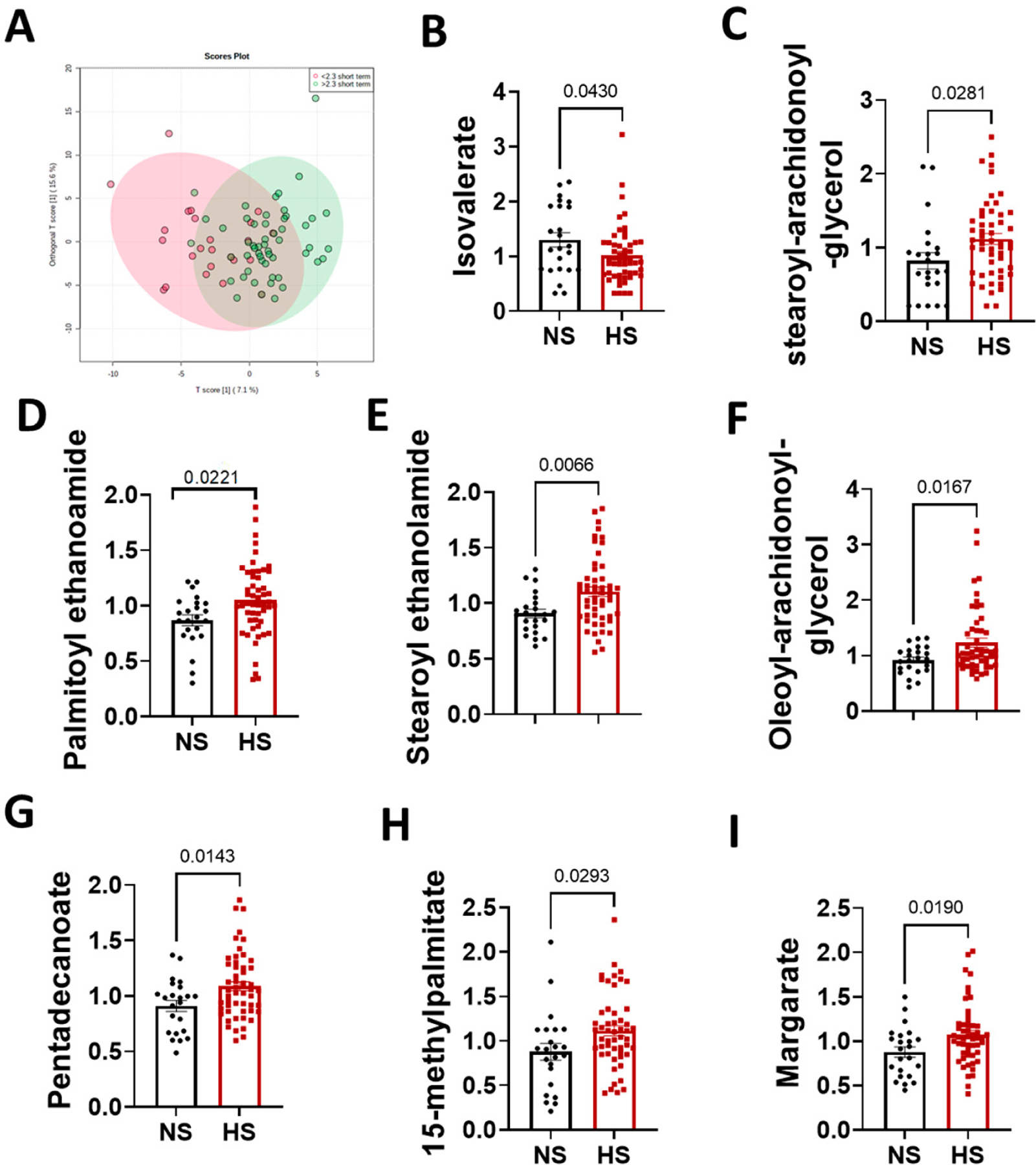
A high-salt diet is associated with changes in plasma fatty acid metabolites. (**A**) Principal component analysis in normal versus high salt fatty acid levels; (**B**) isovalerate; (**C**) stearoyl-arachidonoyl-glycerol; (**D**) palmitoyl ethanolamide; (**E**) stearoyl ethanolamide; (**F**) oleoyl-arachidonoyl-glycerol; (**G**) pentadecanoate; (**H**) 15-methylpalmitate; and (**I**) margate. (*p*-values were determined using 2-tailed unpaired Student’s *t* tests). NS: normal salt (*n* = 23). HS: high salt (*n* = 52).

To investigate the relationship between dietary Na^+^ intake and fatty acids, we conducted an orthogonal partial least squares-discriminant analysis and found distinct clusters of normal and high salt groups (Figure 3A). To evaluate the specific fatty acids that are associated with a high salt diet, we used Student’s *t* test to assess differences between groups in plasma and fecal fatty acids levels. We found eight significantly different fatty acids: isovalerate (Figure 3B) stearoyl-arachidonoyl-glycerol (Figure 3C), palmitoyl ethanolamide (Figure 3D), stearoyl ethanolamide (Figure 3E), oleoyl-arachidonoyl-glycerol (Figure 3F), ceramide; pentadecanoate (Figure 3G), 15-methylpalmitate (Figure 3H) and margarate (Figure 3I).

**Figure 4.**
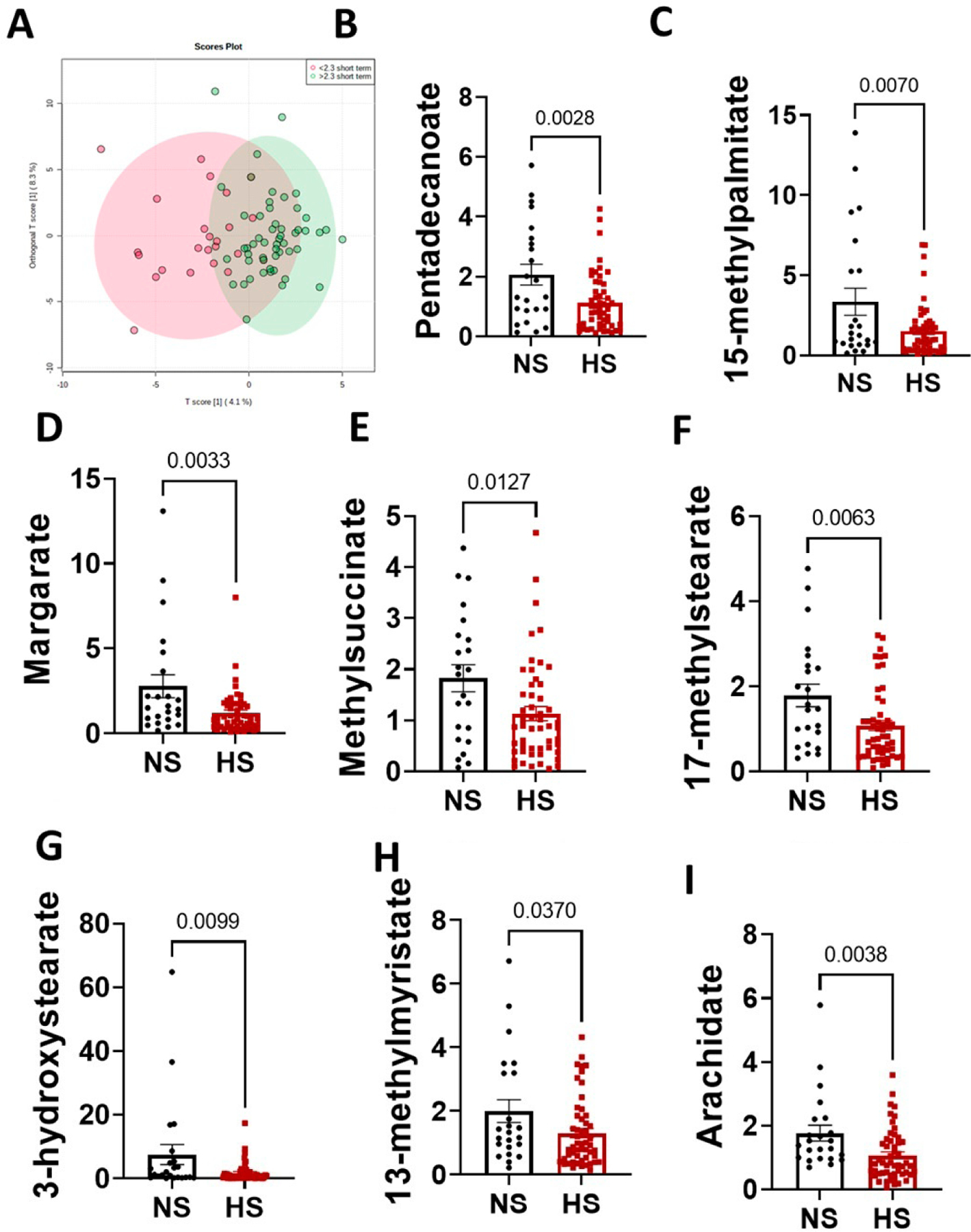
A high-salt diet is associated with changes in fecal fatty acids metabolites. (**A**) Principal component analysis in normal versus high salt fatty acid levels; (**B**) pentadecanoate; (**C**) 15-methylpalmitate; (**D**) margarate; (**E**) methylsuccinate; (**F**) 17-methylstearate; (**G**) 3-hydroxystearate; (**H**) 13-methylmyristate; and (**I**) arachidate. (*p*-values were determined using 2-tailed unpaired Student’s *t* tests). NS: normal salt (*n* = 23). HS: high salt (*n* = 52).

The relationship between high blood pressure and short-chain fatty acids is established [21–23]. However, the dysregulation of fat absorption in the small intestine due to conditions such as gut intestinal mucosa inflammation can lead to changes in other types of fatty acids. We previously demonstrated that high salt and hypertension are associated with inflammation and dysbiosis. Similar to plasma samples, fecal fatty acids from normal and high salt groups clustered distinctly (Figure 4A). In this study, we found no differences in short-chain fatty acids but eight significantly decreased fecal fatty acids. Pentadecanoate (Figure 4B), 15-methylpalmitate (Figure 4C), margate (Figure 4D), methylsuccinate (Figure 4E), 17-methylstearate (Figure 4F), 3-hydroxysterate (Figure 4G), 13-methymyristate (Figure 4H) and arachidate (Figure 4I) were significantly decreased in feces from participants in the high salt group compared to those consuming a normal salt diet.

### 3.3. Sex Differences in the Arachidonic Acid Pathway

**Figure 5.**
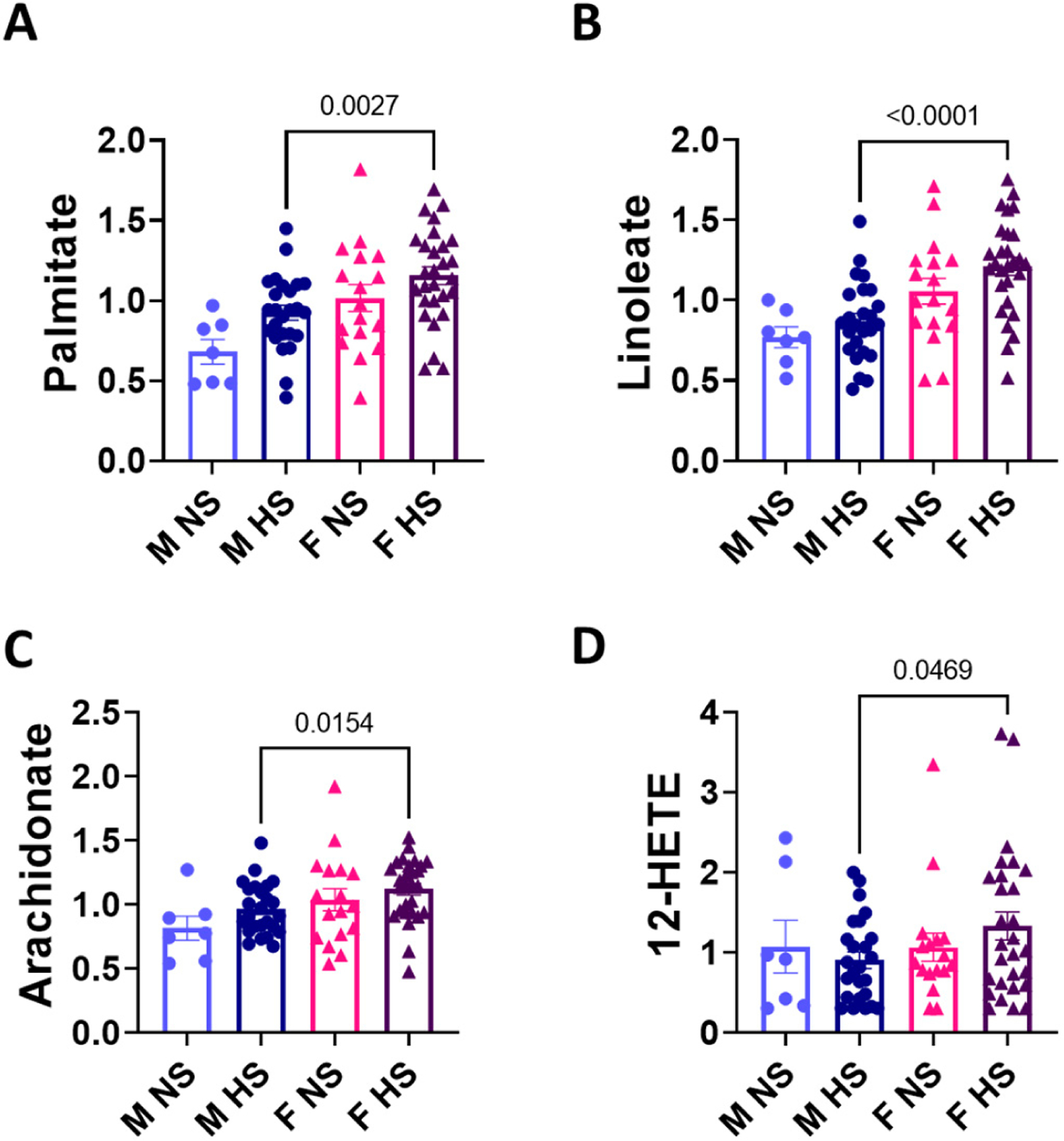
Sex differences in metabolites related to arachidonic acid signaling. (**A**) Palmitate, (**B**) Linoleate, (**C**) Arachidonate and (**D**) 12-HETE in men and women on a normal or high salt diet. M: male; F: female; NS: normal salt; HS: high salt; 12-HETE: 12-Hydroxyeicosatetraenoic acid. *p*-values were determined using 2-tailed unpaired Student’s *t* tests. M NS: men normal salt (*n* = 7); M HS: men high salt (*n* = 24); F NS: women normal salt (*n* = 16); and F HS: women high salt (*n* = 28).

We evaluated sex differences among fatty acids. No sex differences in arachidonic acid metabolites were observed between men and women consuming a normal salt diet. We found that women on a high Na^+^ diet have high levels of palmitate (Figure 5A), linoleate (Figure 5B), arachidonate (Figure 5C), and 12-HETE (hydroxyeicosatetraenoic acid, Figure 5D) suggesting a role of arachidonic acid metabolism in sex differences of Na^+^ response.

### 3.4. Elevated Sodium-Induced Changes in Genes Related to Fatty Acids Signaling in Monocytes Isolated from Women

**Figure 6.**
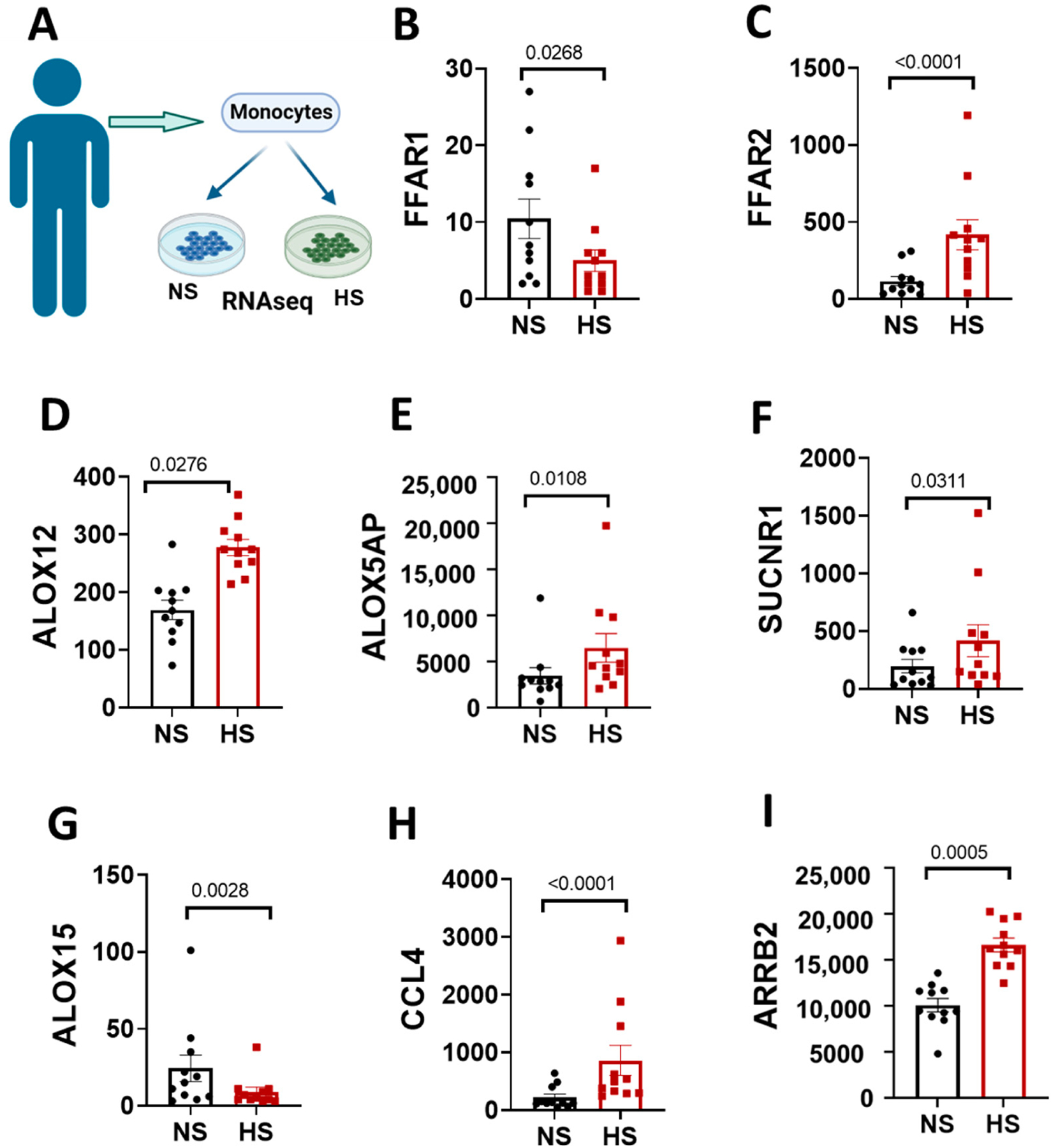
Effect of salt on human monocyte RNA transcriptome. (**A**) Human monocytes were isolated from 11 subjects and treated with normal salt (150 mM NaCl) or high salt (190 mM NaCl) in vitro. (**B**) FFAR1, (**C**) FFAR2, (**D**) ALOX12, (**E**) ALOX5AP, (**F**) SUCNR1, (**G**) ALOX15, (**H**) CCL4 and (**I**) ARRB2 show RPKM for selected receptors and chemokines. Adjusted *p*-values (q) are shown in (**E**,**F**) using a false discovery rate (FDR < 0.05). *p*-values were determined using 2-tailed unpaired Student’s *t* tests, *n* = 11.

The association between high salt and fatty acids signaling in inflammatory pathways is less understood. Using RNAseq (Figure 6A), we investigated changes in fatty acids receptor gene expression in human monocytes that were exposed to high salt in vitro. FC (edgeR) analysis showed that the free fatty acid receptor 1 (FFAR1, Figure 6B) gene was significantly decreased in monocytes treated with high salt, whereas FFAR2 (Figure 6C) was higher compared to monocytes treated with normal salt. Findings from the global metabolic analysis implicated the role of arachidonic acid signaling in response to high salt. Thus, we interrogated whether high salt affects genes related to arachidonic acid signaling in antigen presenting cells. Interestingly, we found ALOX12 (Figure 6D), ALOX5AP (Figure 6E) and SUCNR1 (Figure 6F) to be increased while ALOX15 (Figure 6G) was decreased in human monocytes in response to high salt treatment. The activation of fatty acid receptors is linked to inflammation. Genes that encode for the chemokine ligands 4 CCL4 (Figure 6H) and the lymphocyte chemotaxis regulator β-arrestin 2, arrb2, were also increased after high salt (Figure 6I).

## 4. Discussion

Evidence from clinical trials suggests that women have a higher responsiveness of blood pressure to salt intake regardless of age and hypertension status. In this study, we conducted a metabolomic analysis of men’s and women’s plasma and stool samples to identify potential metabolic players in sex differences and blood pressure responses to high salt. We found that excess Na^+^ is associated with fatty acid metabolism dysregulation. We identified that the arachidonic acid signaling pathway may play a role in sex differences and blood pressure response to high salt.

Although hypertension is more prevalent in men than in premenopausal women, its associated mortality is higher in women [1]. The composition of the gut microbiota is different between men and women at various taxonomic levels. Koliada et al. showed that women have higher Firmicutes and Actinobacteria but lower Bacteroidetes than men. Genera that are associated with health outcomes include Ruminococcus and Bifidobacterium [24,25]. The microbial sex differences are even more pronounced with increasing body mass index [26]. The current understanding of the mechanistic role the gut microbiome contributes to disease is through the various bacteria-derived metabolites and their physiological effects. We have previously shown the association between gut microbiota dysbiosis, hypertension and a high salt diet [8].

Studies indicate that men have higher visceral adipose tissue and are at a greater risk for developing hypertension than premenopausal women [27]. Similarly, men in our cohort had a higher body mass index and larger waist circumference than premenopausal women (Table 1), which may contribute to the development of hypertension. An enlarged waist suggests increased visceral adiposity, which may contribute to hypertension through mechanisms including the activation of the renin-angiotensin-aldosterone system, activation of the sympathetic nervous system, and physical compression of the kidneys [28].

Sex hormones are established crucial players in sex differences in hypertension, but their role in SSBP is less understood. Epidemiologic data show that women lose protection against other forms of hypertension after menopause, which is attributed to the reduction in estrogen [29,30]. Interestingly, ovariectomy abolishes the sex differences in salt-sensitive hypertension in rodents, implicating the role of estrogen [31]. We postulate that the response would be more pronounced in postmenopausal women, as salt sensitivity is reported to inversely correlate with circulating ovarian hormones [32].

Untargeted metabolomic analysis identified an inverse association between amino acids like histidine and salt sensitivity [33,34]. It is less understood whether these sex differences inherently exist outside the context of a high Na^+^ diet. The SmartPanel analysis of plasma metabolites (Figure 2A), not classified by salt intake, revealed sex differences predominantly in the nitrogen, benzoate, histidine, and tyrosine metabolism. Some of these metabolites are known to affect kidney function and blood pressure. Hippurate has a role in microbial degradation and hepatic function and is used as a measure of renal clearance to assess effective renal plasma flow [35,36]. 3-Hydroxyphenylacetic is a flavonoid metabolite that has been shown to potentially reduce blood pressure and vascular relaxation through nitric oxide [37]. Others found that kynurenate, which decreases blood pressure in mice, was higher in females than males [38]. Histidine and tyrosine possess antihypertensive properties, suggesting a potential contribution to premenopausal women’s protection against hypertension [39,40]. Our study found that histidine, glutamine and tyrosine were higher in women than men, suggesting a potential role in the sex differences observed in hypertension, and future studies will investigate their potential contribution to salt-sensitive hypertension. Our findings suggest that the arachidonic acid signaling pathways may play a role in pathophysiology and sex differences and blood pressure response to salt in individuals consuming excess dietary Na^+^. We found that, in addition to linoleate, arachidonic acid and 12-HETE were also significantly higher in women who consumed a high-salt diet (Figure 5). Our results implicate a role of arachidonic acid signaling in the reported high blood pressure response to a high salt diet in women than in men, potentially through inflammatory pathways. High salt-induced hypertension causes the increased accumulation of highly reactive arachidonic acid peroxidation products called Isolevuglandins in dendritic cells [8], promoting T cell activation and the release of cytokines leading to vascular dysfunction, kidney damage, and hypertension [41].

Our results align with previous studies that identified branched-chain amino acids, sugar, and fatty acid metabolism as the top pathways in hypertensive humans [42,43]. These studies, however, did not investigate the effects of a high salt diet or sex differences. Interestingly, the Sodium Feeding Study examined the effect of dietary Na^+^ intake on metabolites within the Dietary Approaches to Stop Hypertension (DASH) and identified fatty acid metabolism as the top pathway associated with Na^+^ intake [44]. Other reports implicate changes in diacylglycerol lipids changes in response to a DASH diet [45]. Similarly, we found stearoyl-arachidonoyl-glycerol and oleoyl-arachidonoyl-glycerol among fatty acids metabolites that were changed in high salt, like in previous reports [46]. Most clinical studies on salt sensitivity involve dietary approaches that span weeks of intervention and more controlled Na^+^ intake measurements. We obtained dietary Na^+^ information based on short-term questionnaire data and acknowledge the limitation of the approach as it may cause underestimation of the salt intake. The current study identified metabolites related to blood pressure changes to high salt and sex differences. These intriguing results will be examined further mechanistically in salt-sensitive men and women. Future studies will recruit and phenotype participants by salt sensitivity, which will be determined using the rigorous Weinberger protocol [47]. Additionally, we will use gold standard dietary salt intake approaches including 24 h urinary Na^+^ excretion.

Circulating monocytes can enter and re-emerge as activated mon from tissues and contribute to blood pressure elevation and renal and vascular damage [48–50]. High Na^+^ activates human monocytes to an antigen presenting phenotype, leading to increased cytokine production and T cell activation [7]. Evidence strongly supports that immune cells such as monocytes can sense Na^+^ activation to contribute to inflammation. Our metabolomics data demonstrated dysregulation in fatty acid pathways, but the specific mechanisms are not well understood. Thus, we assessed potential mechanistic players linking fatty acid metabolism, Na^+^ and inflammation using RNA sequencing in human monocytes treated with high salt. The activation of free fatty acids receptor 1 (FFR1) by fatty acids is a protective mechanism, and we found that high salt downregulated its gene in human monocytes in vitro [51]. Linoleate is a ligand for FFAR1 [52] and was upregulated in women on a high salt diet compared to men. Linoleate is a precursor to arachidonic acid, a well-known player in cardiovascular disease and inflammation [53]. We observed (Figure 6) changes in genes that encode for enzymes of the arachidonic acid pathway, including arachidonate 12-lipoxygenase (ALOX12), arachidonate 5-lipoxygenase activating protein (ALOX5AP), and arachidonate 15-lipoxygenase (ALOX15) in human monocytes. The ALOX15 gene, which suppresses inflammatory pathways TNF-α and NF-κb [54,55], was downregulated in monocytes treated with high salt in our study. On the other hand, ALOX5AP and ALOX12 increased, and they are known to drive inflammation and oxidative stress [56,57]. 12-lipoxygenase produces 12-hydroxyeicosatetraenoate (12-HETE) and is implicated in inflammation and essential hypertension [58–60]. The variability in the ALOX5 and ALOX5AP genes is associated with carotid intima-media thickness, a marker of carotid atherosclerosis, in patients with type 2 diabetes mellitus [61].While these results suggest an effect of high salt on genes that encode for proteins related to arachidonic acid signaling, they do not provide information on which ones are important in sex differences as it pertains to salt sensitivity.

Our initial study indicated that a high salt diet alters the gut microbiome in humans, evidenced by an increase in Firmicutes, Proteobacteria, and genus *Prevotella*. In this study, we used a metabolomics approach to uncover novel players in the mechanisms and sex differences of the salt sensitivity of blood pressure. These findings provide potentially novel insights into metabolic changes underlying gut dysbiosis and inflammation in the salt sensitivity of blood pressure. Future studies will investigate the direct mechanistic link between high salt and the role of the gut microbiome in regulating fatty acid metabolism in hypertension.

## Figures and Tables

**Table 1.** Demographic and clinical characteristics of the subjects by short-term sodium intake.

	Female (*n* = 44)	Male (*n* = 31)	Test Statistic
**Age (years)**	28.68 ± 7.953	32.71± 9.324	*p* = 0.0481
**Race**			
Asian	4		
Black	16	4	
White	24	27	
**Body Mass Index (kg/m** ^**2**^ **)**	24.27 ± 6.17	27.14 ± 5.57	*p* = 0.0423
**Waist circumference (cm)**	76.76 ± 13.37	91 .69 ± 15.18	*p* < 0.0001
**Hip circumference**	98.96 ± 12.23	103.4 ± 10.35	*p* = 0.1053
**Average DBP**	67.31 ± 8.075	72.76 ± 9.24	*p* = 0.0076
**Average SBP**	110.80 ± 10.92	122.8 ± 11.50	*p* < 0.0001
**Average MAP**	80.25 ± 14.84	89.44 ± 9.41	*p* = 0.0033
**Heart Rate**	71.48 ± 7.56	70.35 ± 8.41	*p* = 0.5476
**Daily Na**^**+**^ **intake (g)**	2.74± 1.05	3.25 ± 1.17	*p* = 0.0582
